# Quantitative patterns of vertical transmission of deformed wing virus in honey bees

**DOI:** 10.1371/journal.pone.0195283

**Published:** 2018-03-29

**Authors:** Esmaeil Amiri, Per Kryger, Marina D. Meixner, Micheline K. Strand, David R. Tarpy, Olav Rueppell

**Affiliations:** 1 Department of Biology, University of North Carolina at Greensboro, Greensboro, NC, United States of America; 2 Department of Agroecology, Aarhus University, Slagelse, Denmark; 3 Department of Entomology & Plant Pathology, North Carolina State University, Raleigh, NC, United States of America; 4 Bieneninstitut Kirchhain, Landesbetrieb Landwirtschaft Hessen, Kirchhain, Germany; 5 Life Sciences Division, U.S. Army Research Office, Research Triangle Park, NC, United States of America; University of California San Diego, UNITED STATES

## Abstract

Deformed wing virus (DWV) is an important pathogen in a broad range of insects, including honey bees. Concordant with the spread of *Varroa*, DWV is present in the majority of honey bee colonies and can result in either low-level infections with asymptomatic bees that nonetheless exhibit increased colony loss under stress, or high-level infections with acute effects on bee health and viability. DWV can be transmitted vertically or horizontally and evidence suggests that horizontal transmission via *Varroa* is associated with acute symptomatic infections. Vertical transmission also occurs and is presumably important for the maintenance of DWV in honey bee populations. To further our understanding the vertical transmission of DWV through queens, we performed three experiments: we studied the quantitative effectiveness of vertical transmission, surveyed the prevalence of successful egg infection under commercial conditions, and distinguished among three possible mechanisms of transmission. We find that queen-infection level predicts the DWV titers in their eggs, although the transmission is not very efficient. Our quantitative assessment of DWV demonstrates that eggs in 1/3 of the colonies are infected with DWV and highly infected eggs are rare in newly-installed spring colonies. Additionally, our results indicate that DWV transmission occurs predominantly by virus adhering to the surface of eggs (transovum) rather than intracellularly. Our combined results suggest that the queens’ DWV vectoring capacity in practice is not as high as its theoretical potential. Thus, DWV transmission by honey bee queens is part of the DWV epidemic with relevant practical implications, which should be further studied.

## Introduction

Ongoing research into the causes of the honey bee health crisis has led to considerable progress in better understanding of the distribution and effects of many honey bee diseases [1–3]. However, a thorough fundamental understanding of the mechanisms and quantitative aspects of the transmission of most bee pathogens is still lacking. Particularly, honey bee viruses are poorly characterized. Among them, DWV is the most important virus belonging to the family *Iflaviridae* [4]. It is one of several emerging insect RNA viral pathogens that has been detected in a wide range of invertebrate species, including bumblebees, solitary bees, wasps, hornets, ants and hoverflies [5–8]. DWV has been detected in all honey bee castes and sexes (queens, workers, and drones) and all developmental stages (sperm, eggs, larvae, and pupae [4, 9]). Infection with DWV has been reported in honey bee populations as both overt disease or asymptomatic infection in over 50% of colonies and 90% of apiaries [10–12].

In association with the parasitic mite *Varroa destructor*, which vectors DWV horizontally when feeding on honey bees and their larvae and pupae, DWV causes overt disease that leads to colony weakening and mortality worldwide [13, 14]. Disease symptoms, such as deformed wing, shortened abdomens, discoloring, behavioral abnormalities, and reduced lifespan, are most commonly reported in adult drones and workers that became infected during development [4, 11]. In symptomatic individuals, the virus is prevalent in all body parts, but accumulates especially in the epithelial cells of digestive tract, shedding large amount of virus particles into the lumen [15]. In addition, DWV can accumulate in the testes, mucus glands, and seminal vesicles of drones [15], while in queens the highest DWV titers are found in the ovaries [15] but see [16]. DWV infection may also cause extreme cases of ovarian degeneration in queens [17]. This accumulation in reproductive tissues might represent an adaptive predisposition for DWV to enhance its vertical transmission.

Modern apicultural practices aim to minimize disease prevalence in colonies [18], however, this practice and the symbiosis [19] between DWV and *Varroa* selects for particularly virulent DWV strains and leads to colony death [10, 14, 19]. Although DWV can be present in individuals and colonies that are asymptomatic, these asymptomatic DWV infections are associated with higher colony winter mortality [20, 21], suggesting damage adult individuals that goes unnoticed upon visual inspection [22].

In addition to the important horizontal transmission by the *Varroa* mite [23], DWV is also transmitted horizontally among adult bees through common visits to flowers, pollen, trophallactic activities, hygienic behavior, grooming and cannibalism [24, 25]. Specifically, glandular secretions of infected nurse bees can infect young larvae and thus transmit DWV to the next generation [26]. DWV-infected queens have theoretically the highest vertical vectoring capacity because they produce all colony offspring [27], but the practical relevance and quantitative details about this vertical transmission are only beginning to emerge [28]. Queens can readily be infected by venereal transmission in the laboratory [29, 30] and under field conditions [31] by DWV infected drones that are able to reach drone congregation areas [32]. Venereal transmission of DWV to queens can spread throughout the queen’s body and damage her interior organs [29, 31]. The virus can reach the ovaries and spermatheca and transmits vertically to the next generation [29, 30, 33]. This vertical transmission is typically accompanied by the absence of disease symptoms and can result in long term persistence of a DWV infection in the population. However, asymptomatic DWV infections can give rise to overt disease symptoms when colonies become stressed or encounter certain environmental conditions [34, 35].

The vertical transmission of DWV by queens has been studied to some extent, but many questions remain. Previous studies have demonstrated that vertical transmission of DWV occurs under laboratory [29, 30] and field conditions [28]. These studies found that only a portion of infected queens give rise to infected eggs, but the reasons for the variable transmission are unclear [29, 30]. More quantitative studies that combine the assessment of queens and resulting eggs are needed to test the hypothesis that the level of queen infection is the primary determinant of vertical transmission. The variability within previous studies [29, 30] also precludes conclusions on how efficient and widespread vertical DWV transmission is under field conditions. Data from a survey in Belgium suggest that 40% of eggs produced by commercial queens are infected with DWV [28]. Due to the strong seasonal and geographic variability of DWV [34, 36], the generality of this finding is unclear, even though the study includes multiple queen breeders [28], but the inclusion of 11 queen breeders in this survey limits the within-operation sample size and prevents a satisfactory assessment of within-operation heterogeneity of vertically transmitted DWV.

The mechanisms of vertical DWV transmission have been studied previously, but some contradictory findings prevent a conclusive understanding of how DWV is passed on to the next generation in detail. Fertilized and unfertilized eggs seem to be infected at similar level, indicating that the fertilization process itself is not necessary for DWV transmission [30]. However, it is unclear whether DWV is incorporated into the eggs (transovarial transmission) or passed on via surface contamination of the eggs (transovum transmission). A study of PBS-washed eggs did not detect any DWV [9] suggesting transovum transmission, while a simultaneous study of bleach-washed eggs indicated transovarial transmission [33].

Here, we performed a series of three studies of the vertical transmission of DWV to further our understanding of *Varroa*-independent transmission pathways of DWV in honey bees. We compared the DWV titers of eggs produced by queens with quantitative information on the variable DWV levels in their various tissues. Secondly, we surveyed a large population of commercial hives from one beekeeper in the southeastern US at the beginning of the beekeeping season to study the incidence and quantitative heterogeneity of egg contamination by DWV. Thirdly, we performed an additional study to differentiate among the three possible vertical transmission pathways from queens to new offspring: infection by sperm, transovarial, or transovum transmission.

## Material and methods

### Experimental 1: Quantitative DWV transmission study

Thirty young Buckfast queens were produced in a colony with minimal *Varroa*- and DWV levels following the standard procedure [37] by a professional queen producer in the Reerslev, Denmark (55° 33' 21.1788'' N 11° 23' 25.4256'' E). Before grafting, the donor and rearing colonies had been confirmed to be treated against *Varroa* mites and free of most common bee viruses as described previously [38]. The queens were introduced to mating hives containing 250–300 *Varroa*-free worker bees. These mating hives were placed in a mating station (Flakkebjerg, Denmark, 55° 19' 31.278'' N 11° 23' 28.6188'' E) surrounded by drone provider colonies that had not been treated against *Varroa* mites for the past three years and consequently furnished drones with relatively high DWV infections for the experimental queens to mate with [31]. After mating, the queens developed DWV infections that were highly correlated among different body parts of each queen (head, thorax, abdomen, ovary, spermatheca, and sperm) and differed strongly between individual queens (DWV titers ranging from 0 to >10^10^) [31]. Three weeks after the onset of oviposition, one batch of 50 freshly laid eggs was collected from just constructed waxcomb into a micro-centrifuge tube from each of 25 reproductive queens. These samples were immediately stored at -80°C until RNA extraction. Micro-pestles (Eppendorf) were used to homogenize the egg samples, and total RNA was extracted from each sample using NucleoMag® 96 RNA Kit (Macherey-Nagel) on a Kingfisher Magnetic Extractor following the manufacturer’s guidelines. RNA concentration and purity were measured using a Nanodrop ND-1000 spectrophotometer (Thermo Scientific) and total RNA concentration was adjusted to 20 ng/μL with molecular grade water (Fisher Scientific). The RNA was stored at -80°C for further use.

### Experiment 2: Survey of commercial population for virus transmission in eggs

A commercial population of 85 colonies headed by Italian-queens in five apiaries were surveyed for the DWV content of eggs at the beginning of beekeeping season with the kind permission of beekeeper. The apiaries belonged to a single migratory beekeeper near Mebane (LEE’s BEES Inc, North Carolina, USA). These apiaries were in 10 km distance of the main honey bee station (approximate location 36° 7' 6.4416'' N 79° 15' 13.2768'' W). The colonies were sampled on the 19^th^ - 28^th^ of April 2016, approximately three weeks after colony establishment from 3-lb packages. This early sampling time was chosen to provide a baseline estimate of DWV transmission through queens prior to the seasonal build-up of *Varroa*. Fifty freshly laid eggs were carefully collected from worker size cells of newly-produced comb and transferred into one micro-centrifuge tube per colony. The samples were transported on ice back to the laboratory where they were stored at -80°C until RNA extraction. Eggs in each micro-centrifuge tube were homogenized using micro-pestles (Fisher Scientific) and total RNA was extracted with a standard Trizol™ protocol [39]. The RNA concentration and purity were measured, adjusted and stored as above until further processing.

### Experiment 3: Characterizing the mechanism of vertical transfer of DWV

Based on the results of the second experiment, five queens that laid DWV-infected eggs were transferred from the commercial beekeeping operation in Mebane, NC, to the UNCG apiary in Greensboro, NC (36° 5' 55.7448'' N 79° 53' 21.4116'' W), for further study. Queens were introduced to mini-hives (Styrofoam™ mating nucs, Mann Lake USA) with empty newly-produced worker and drone cells to induce each queen to lay simultaneously fertilized and unfertilized eggs. Three samples of 50 eggs were collected from each queen in micro-centrifuge tubes: 50 eggs from worker size cells and two batches of 50 eggs from drone size cells. One batch of drone eggs remained unmanipulated, while the other one was surface-sterilized by immersion in 5% bleach solution for five minutes followed by three rinses in sterile water [2, 33]. All samples were stored at -80°C until RNA extraction. The total RNA for each sample was extracted, its concentration and purity were measured, adjusted and stored as above until further processing.

### cDNA synthesis, qPCR assays and data analysis

Using the stored RNA from all three experiments, a two-step quantitative qPCR assay was carried out to quantify the DWV viral load in the samples. For each sample, cDNA was synthesized using the High Capacity cDNA Reverse-Transcription Kit (Applied Biosystems). RNA template (10 μL) with a final concentration of 20 ng/μL was added to10 μL of the provided cDNA master mix, followed by an incubation period as recommended by the manufacturer: 10 min at 25°C, 120 min at 37°C and 5 min at 85°C. The cDNA solution was then diluted 10-fold in molecular grade water to serve as template in subsequent qPCRs to quantify DWV and other targets using unlabeled primers and SYBR Green DNA binding dye (Applied Biosystems). Quantification was performed in duplicate and in a reaction volume of 12μL for the samples in Experiment 1 and 20μL for the samples in Experiments 2 and 3. Final primer concentrations of 0.4μM were used. DWV primers used in this study quantify DWV type A [40]. The reference genes β-Actin and RPS5 were used as an internal control and for relative quantification of DWV using the ΔCt method [2]. A positive control was run in each case, and RNase-free water was added as template for a No Target Control (NTC), and a No Reverse Transcriptase (NRT) control served as an additional negative control [41]. The thermal cycling conditions using a viiA™7 apparatus (Applied Biosystems) for Experiment 1 and StepOnePlus™ (Applied Biosystems) for Experiment 2 and 3 were 10 min at 95°C, followed by 40 cycles consisting of a denaturing stage at 95°C for 15 s and as annealing/extension stage at 60°C for 1 min. Fluorescence measurements were taken at the end of each cycle. This procedure was followed by a final melt-curve dissociation analysis to confirm the specificity of the products. The primers used in this study (Table 1) have previously been validated to detect the intended targets and are commonly used in honey bees [21, 42–46]. Samples were deemed positive for a target if their melting temperature was similar to the melting temperature of the positive controls and a C_t_ value of 35 or lower was recorded. Our virus survey in the second experiment also screened for Sacbrood virus (SBV) and the Acute Bee Paralysis Virus complex (AKI: Acute Bee Paralysis Virus, Kashmir Bee Virus, and Israeli Acute Bee Paralysis Virus) to assess the co-occurrence of these viruses with DWV.

**Table 1:** Primers used to establish standard curves and analyze samples.

Target	Primers name		Primer sequence	Product size(bp)	Reference
**DWV**		DWV-fwd DWV-rev	5’-TTCATTAAAGCCACCTGGAACATC 5’-TTTCCTCATTAACTGTGTCGTTGA	136bp	[42]
**DWV**		F-DWV R-DWV	5’-GGATGTTATCTCCTGCGTGGAA 5’-CTTCATTAACTGTGTCGTTGATAATTG	69bp	[44]
**SBV**		SBV-F434 SBV-R503	5’-AACGTCCACTACACCGAAATGTC 5’-ACACTGCGCGTCTAACATTCC	70bp	[47]
**AKI**		F-AKI R-AKI	5’-CTTTCATGATGTGGAAACTCC 5’-AAACTGAATAATACTGTGCGTA	100bp	[45]
**RPS5**		RpS5-F RpS5-R	5’-AATTATTTGGTCGCTGGAATTG 5’-TAACGTCCAGCAGAATGTGGTA	115bp	[43]
**β.Actin**		F-β-Actin R-β-Actin	5’-TGCCAACACTGTCCTTTCTGGAGGT 5’- TTCATGGTGGATGGTGCTAGGGCAG	96bp	[21]

Virus loads in each sample were quantified using absolute quantification methods based on standard curves obtained through serial dilutions of known amounts of amplicons as described before [21]. The successful amplification of reference genes (β-Actin and RPS5) was used to confirm the integrity of samples throughout the entire procedure, from RNA extraction to qPCR. The reference genes were also used for relative quantification of virus titers. Results of relative and absolute quantification did not significantly differ, thus only absolute values (copy number / μL) are presented. To improve data compliance with parametric assumptions, raw data were log_10_ transformed where parametric analysis was necessary [39]. Data analysis and visualization were performed using Excel and “R”, version 3.1.3.

## Results

### Experiment 1: Quantitative DWV transmission study

**Fig 1:**
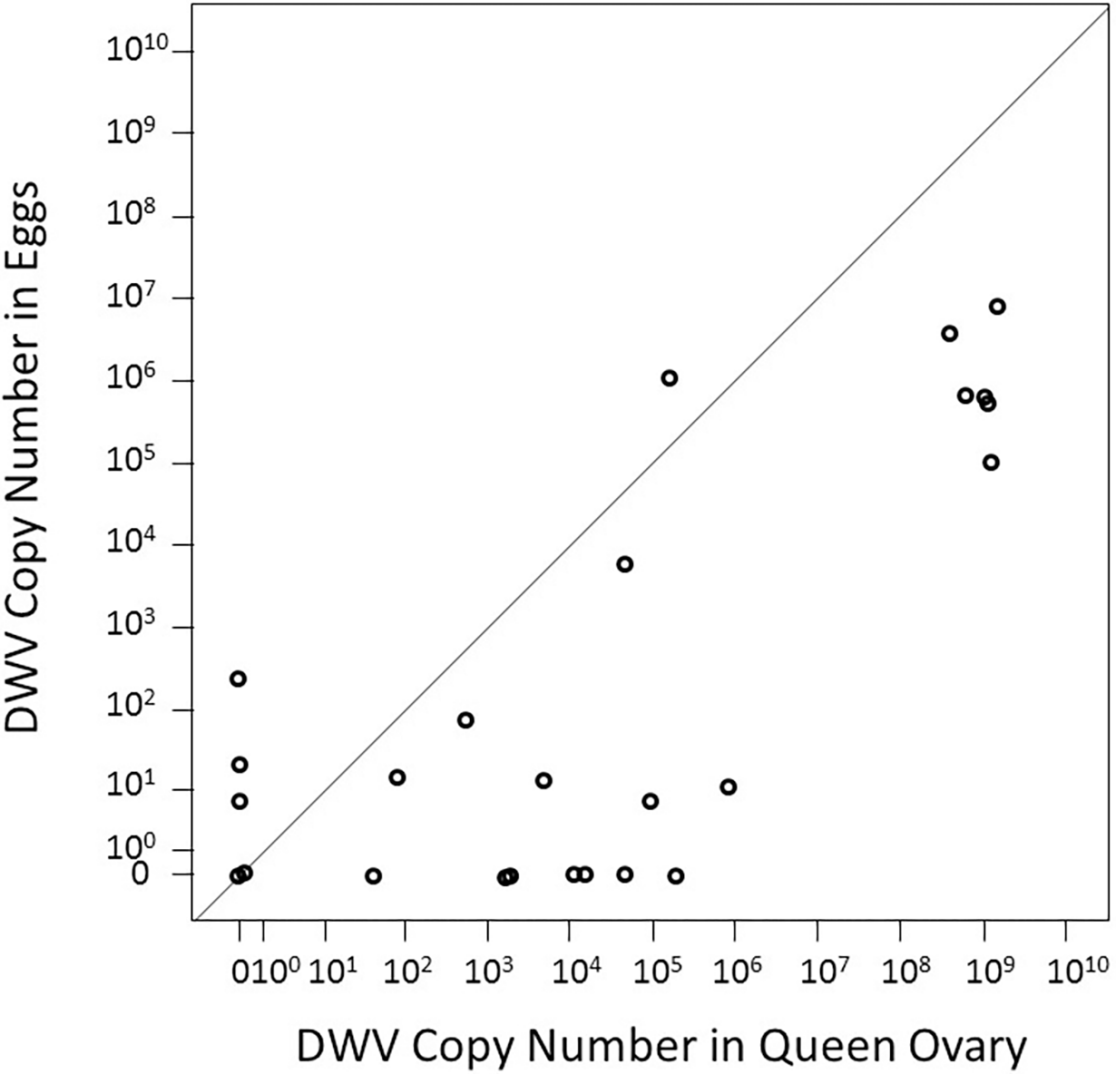
DWV copy number (copies / μL) in eggs and ovaries of experimental queens. A positive relation between a queen’s ovaries and the eggs she produced existed despite considerable variation. Eggs contained less DWV than the ovary in most cases (data points falling below the diagonal line), indicating an imperfect vertical transmission. Although no clear infection threshold for vertical DWV transmission was indicated, all highly infected queens transmitted DWV while queens with lower DWV titers commonly failed to transmit detectable amounts of DWV to their eggs.

DWV titers in the experimental queens were variable among queens [31] and titers were highly correlated in the different body parts (Spearman’s R > 0.89, n = 25, p < 0.001). The DWV titers in eggs produced by these queens also varied widely and were significantly correlated to DWV titers in the ovary (Rs = 0.56, n = 25, p = 0.004) and all other body parts of individual queens. Nine egg samples contained no DWV, although the corresponding ovary from which they originated was infected with DWV (titers ranged from 58 to 1.9 × 10^5^ copies per μl). The six most highly-infected queens all produced eggs containing considerable amounts of DWV (1 × 10^5^–6.7 × 10^7^ copies per μl). Four egg samples exhibited higher DWV titers than the respective queen’s ovary, while in 19 cases eggs contained less DWV than the respective ovary (Fig 1). Thus, eggs overall contained significantly less DWV than the ovary (sign test: p < 0.05). β-Actin was consistently amplified in all samples with an average Ct value of 16.0 ± 0.9 (S.D.).

### Experiment 2: Survey of commercial population for virus transmission in eggs

DWV was detected in 27 out of 85 egg samples from unique colonies, including only one sample with very high titers (Table 2). The Sacbrood Virus (SBV) was detected in 38% (32/85) of samples, but we could not detect any of Acute Bee Paralysis Virus complex. Double-infections with DWV and SBV occurred in 8 colonies, which was not significantly different from what was expected by chance (Fisher’s exact test: p = 0.21). Neither the prevalence of DWV and SBV (Fisher’s exact test: p = 0.71 and p = 0.28, respectively), nor the intensity of infection (Kruskal-Wallis tests of virus titers: p = 0.59 and p = 0.103, respectively) varied significantly among the five apiaries. Amplification of the RPS5 control was consistent among all samples (average Ct value of 19.7 ± 2.0 (S.D.)) and indicated no major technical variation in sample quality.

**Table 2:** DWV and SBV content of 85 commercial, early-season colonies.

Classification	Virus titer (copies / μL)	No. Samples DWV	No. Sample SBV
**No infection**	0	58	53
**Low infection**	0 < C < 10^3^	10	25
**Medium infection**	10^3^ ≤ C < 10^7^	16	7
**High infection**	C ≥ 10^7^	1	0

### Experiment 3: Characterizing the mechanism of vertical transfer of DWV

**Fig 2:**
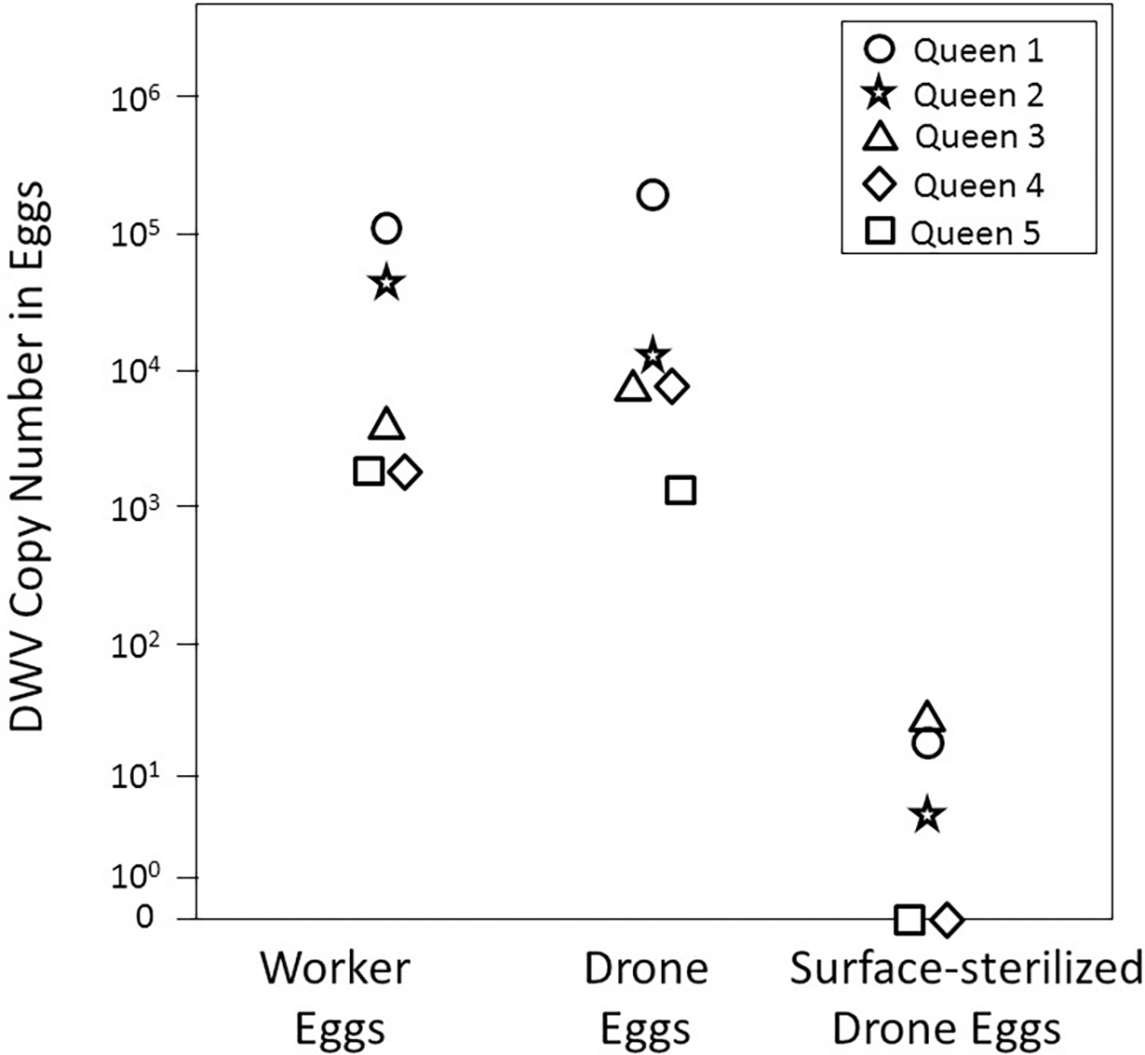
Comparison of DWV titers in worker, drone and surface-sterilized drone eggs. Significantly lower (p < 0.001) DWV titers in the surface-sterilized samples indicate that most DWV adheres to eggs externally, indicating that the predominant vertical DWV transmission pathway is transovum. Each data point represents 50 eggs.

Considerable amounts of DWV were detected in worker and drone eggs without surface sterilization (1.9 × 10^3^–1.1 × 10^5^ copies per μL and 2.3 × 10^3^–2.1 × 10^5^ copies per μL, respectively). Surface sterilization of drone eggs resulted in much lower DWV titers (0–31 copies per μL). Overall, the groups were significantly different (Repeated Measures ANOVA: F_(2,8)_ = 103.7, p<0.001; Fig 2) due to the significantly lower DWV level in surface sterilized drone eggs than in non-sterilized drone (Tukey’s posthoc test: p<0.001) and worker (p<0.001) eggs. RPS5 amplified consistently without significant group differences (Ct-values of 20.9 ± 2.4, 19.7 ± 0.8 and 18.6 ± 1.2 for worker, drone, and sterilized drone eggs, respectively).

## Discussion

Our results quantify vertical transmission of DWV from infected queens to the progeny at the individual and population level and indicate that this transmission occurs primarily through virus adhering to the surface of eggs (transovum). The quantitative assessment suggests that this transmission is common but not highly efficient, despite the accumulation of DWV in the queen ovary [15].

Honey bees and other highly social organisms are particularly vulnerable to horizontal disease transmission because they live in physical proximity of one another with frequent contact among individuals [11]. The arrival of *Varroa* mite provides DWV with an effective vectored transmission route, benefitting both mite and virus population growth [13, 19, 48]. However, sociality also entails reproductive division of labor that in turn may facilitates vertical disease transmission because a few reproductive specialists generate the entire next generation. Previous data demonstrated that DWV uses vertical transmission through queens [29, 30, 33], which presumably played a more crucial role in the interaction between DWV and honey bees before the arrival of *Varroa* [12–14].

The quantitative comparisons of DWV titers between queens and their eggs in our first experiment indicate that the transmission of DWV is highly dependent on the infection level of the queen. Thus, determining the DWV titer of eggs enables us to make inferences about the infection level of honey bee queens. In combination with this finding, our population survey of newly established commercial colonies suggests significant variability in the DWV infection level among commercial queens in the same operation that come from the same queen breeder. Presumably, this argument also applies to SBV, which has been reported to co-occur in queens and eggs before [28, 49], but our data do not allow us to draw further conclusions for this virus. Even though the SBV infection levels are lower than those of DWV, the prevalence of the two viruses in our study population is similar and comparable with a recent study in Belgium [28], but lower than SBV prevalence reported from Pennsylvania 12 years ago [49]. The Acute bee paralysis virus complex was not detected in the commercial survey population, but we do not know whether these three viruses were not present in the queens or not effectively transmitted to the surveyed eggs.

Our study indicates that at least the highly infected queens represent a significant long-term colony health risk to the colony by vectoring DWV. Thus, non-invasive methods for virus screening of queens could be developed for identifying and replacing such queens in apicultural practice. Screening batches of eggs early in the season may represent such a tool, although less tedious and cheaper methods would be preferable in practice. A second practically important result of our study is the documented variability in DWV susceptibility and transmission among queens that were treated identically (Experiment 1) or kept under very similar conditions (Experiment 2). Despite the significant correlation between DWV in queens and their eggs, the ratio of queen to egg titers varied dramatically, suggesting different transmission efficiencies. The overall correlation between egg and queen DWV titers in the first experiment indicates that the variable egg titers in the second experiment are most likely due to differences in queen virus titer. The differences in queen virus titers could be explained by venereal infection via their mating partners, but it could also indicate different susceptibility of these queens to DWV. Similar variation among queens was previously found [29]. If the variation in susceptibility and transmission efficiency has a genetic basis, these traits should be integrated into honey bee breeding programs [50].

In contrast to the incontrovertible evidence for vertical transmission of DWV from honey bee queens to their progeny [29, 30], contradictory findings on the details of how DWV is passed on have been reported. Specifically, evidence for both, transovarial and transovum transmission has been reported [9, 33]. Our surface sterilization of egg samples from DWV infected queens reduced the egg DWV titers by over 1000-fold without significantly reducing the level of control gene expression. Therefore, we conclude that >99.9% of DWV is located on the outside of the eggs, suggesting that transovum transmission is predominant. However, we cannot exclude a low level of transovarial transmission. Hatching honey bee larvae may become instantly infected with DWV from the egg shell. No overall consensus exists on whether transovum or transovarial disease transmission is more important in honey bees or insects in general. Discrepancies among studies may arise through differences in the duration of the virus infection or other experimental circumstances, such as maternal age, but the influence of such factors has not yet been sufficiently addressed. In any case, our results suggest that assuming a transovarial mechanism without further evidence (e.g., [49]) should be avoided in any system. Transovum transmission—which does not require DWV to specifically enter the oocyte—is consistent with the notion of DWV as an opportunistic pathogen prior to the arrival of *Varroa* as a vector [33].

The vertical transmission through long-lived, highly reproductive queens may have ensured the persistence of DWV in honey bee populations, selecting for low virulence [12]. The introduction of horizontal vectoring by *Varroa*, particularly in combination with high-density apiculture that facilitates disease transmission among colonies, has presumably altered dominant transmission routes and virulence by selecting particular DWV genotypes [13, 14, 35]. Our study did not contrast these two transmission pathways and their implications for virulence evolution but demonstrates that vertical transmission of DWV persists and is important to consider in epidemiological models and apicultural management. The quantitative aspects of our study highlight the importance of dose and timing of infection, which should be addressed in further, more controlled experiments.

Although extreme polyandry has been shown to be beneficial to colony productivity and survival [51–54], DWV vertical transmission could influence the benefits of and selection for extreme polyandry because multiple mating exposes the queen to more, potentially DWV infected drones. Conversely, vertical transmission is associated with less virulent DWV genotypes, potentially providing some benefit to the colony if super-infection exclusion or similar phenomena exist [55].

## Supporting information

S1 Supporting Data DWV and SBV titers in different experiment. The virus titer for the three experiments are deposited in the supplementary file.
(XLSX)
